# CDKN1A as a potential target for Eltrombopag treatment in ITP and its regulation of the communication between macrophages and transitional B cells in ITP

**DOI:** 10.1007/s00277-025-06436-5

**Published:** 2025-06-14

**Authors:** Shixuan Wang, Mankai Ju, Fancong Kong, Yuhuan Jiang, Yechao Tu, Jingyun Zou, Zhiming Zou, Genmei Tan, Fei Li

**Affiliations:** 1 Center of Hematology, The First Affiliated Hospital, Jiangxi Medical College, Nanchang University, No. 17 Yongwai Zhengjie, Nanchang, Jiangxi 330006 China; 2 Jiangxi Clinical Research Center for Hematologic Disease, Nanchang, Jiangxi 330006 China; 3 Institute of Lymphoma and Myeloma, Nanchang University, Nanchang, Jiangxi 330006 China; 4 Jiangxi Provincial Key Laboratory of Hematological Diseases, The First Affiliated Hospital, Jiangxi Medical College, Nanchang University, Nanchang, Jiangxi 330006 China; 5 State Key Laboratory of Experimental Hematology, Haihe Laboratory of Cell Ecosystem, Institute of Hematology & Blood Diseases Hospital, National Clinical Research Center for Blood Diseases, Chinese Academy of Medical Sciences & Peking Union Medical College, Tianjin, 300020 China; 6 Laboratory Department of Nanchang, University First Affiliated Hospital, Nanchang, Jiangxi 330006 China

**Keywords:** Immune thrombocytopenia, Macrophage, CDKN1A, Transitional B cells, TGF signaling pathway

## Abstract

This study aimed to identify novel biomarkers associated with Eltrombopag response in patients with immune thrombocytopenia (ITP) and to investigate the role of macrophage and transitional B cells in ITP pathogenesis. Differentially expressed genes were identified using the GSE112278 dataset, followed by weighted gene co-expression network analysis (WGCNA) to screen hub genes. Single-cell RNA-seq data from GSE196676 were analyzed using the Seurat package to assess immune cell composition, gene expression, and cell–cell communication. CDKN1A expression was experimentally modulated in RAW264.7 macrophages via siRNA knockdown or plasmid overexpression. Phagocytic function was assessed using CFDA-labeled mouse platelets and F4/80 immunofluorescence staining. Molecular docking was conducted to evaluate the interaction between Eltrombopag and CDKN1A. Through intersection analysis, we identified CDKN1A as a key gene influencing the response of ITP patients to Eltrombopag treatment. Single-cell data analysis revealed a significant increase in the proportion of macrophages in ITP patients, accompanied by downregulation of CDKN1A expression in these macrophages, which was closely associated with macrophage activation and enhanced phagocytic capacity. Functional experiments confirmed that CDKN1A knockdown promoted, while overexpression inhibited, macrophage phagocytosis of platelets. Additionally, cell communication analysis demonstrated that macrophages in ITP patients interact with transitional B cells via the TGFβ signaling pathway. Further analysis revealed that a subset of macrophages performs effector functions by differentiating into specialized subtypes that function independently, without direct interaction with other immune cells. Our study identified CDKN1A as a key regulator of Eltrombopag’s effectiveness in treating ITP. CDKN1A expression was reduced in macrophages of ITP patients and that it interacted with transitional B cells through the TGFβ signaling pathway to promote disease progression. These findings offer new insights into the pathogenic mechanisms of ITP and suggest CDKN1A as a potential therapeutic target for future interventions.

**Supplementary Information:**

The online version contains supplementary material available at 10.1007/s00277-025-06436-5.

## Introduction

Immune thrombocytopenia (ITP) is a prevalent autoimmune disorder characterized by a reduction in platelet count, often associated with bleeding symptoms [1–3]. The pathogenesis of ITP primarily involves the production of antibodies against platelets by the immune system, resulting in platelet destruction [4, 5]. The primary therapeutic goal of ITP treatment is to elevate platelet counts and reduce the risk of bleeding [6]. As such, corticosteroids, intravenous immunoglobulin (IVIG), and anti-D immunoglobulin are commonly used as first-line treatments for ITP [1, 7, 8]. Although corticosteroids remain the standard first-line treatment for both children and adults, existing data do not demonstrate that corticosteroids significantly improve patients’ health-related quality of life or other patient-centered outcomes [1, 9]. In fact, long-term corticosteroid use may negatively impact the health of both children and adults, manifesting as adverse effects such as sleep disturbances, weight gain, and mental health issues [10, 11]. Therefore, despite advances in ITP management, numerous areas still require urgent research to improve treatment outcomes and patients’ quality of life.

Emerging evidence suggests that aging plays a key role in immune system dysfunction, a phenomenon known as immunosenescence [12]. Aging disrupts the immune microenvironment, impairing immune tolerance and leading to aberrant activation of self-reactive T and B cells, thereby intensifying platelet destruction [13, 14]. Simultaneously, aging alters the homeostasis of the bone marrow microenvironment, suppresses megakaryocyte proliferation and maturation, and markedly diminishes platelet production [15]. Dysregulation of cell cycle control also significantly influences the onset and progression of ITP. Megakaryocyte proliferation may be restricted due to abnormal expression of cell cycle regulatory genes, autophagic dysfunction, and cell division arrest, contributing to a further decline in platelet production [16–18]. More importantly, aging and the cell cycle may interact, establishing a vicious cycle. Aging-induced immune abnormalities and bone marrow microenvironment dysfunction may exacerbate cell cycle abnormalities, while cell cycle disruptions may further impair megakaryocyte function and exacerbate immune regulation imbalance. This bidirectional effect not only directly influences platelet production but also exacerbates disease progression by altering the immune microenvironment. Although current studies have identified some associations, the specific molecular mechanisms through which aging, cell cycle abnormalities, and immune imbalance synergistically drive the onset of ITP remain inadequately explored.

In clinical practice, thrombopoietin receptor agonists (TPO-RAs), such as eltrombopag, have emerged as an effective treatment option for patients with refractory ITP by enhancing platelet production through the stimulation of megakaryocyte proliferation and differentiation [19, 20]. A recent clinical study demonstrated that eltrombopag induced a significant early platelet response and reduced the frequency and severity of bleeding events in patients with acute (ndITP), chronic (cITP), and treatment-naïve (pITP) ITP [21]. In addition to promoting platelet production through TPO receptor activation, TPO receptor agonists (TPO-RAs) may also influence immune regulation. Studies of ITP patients who responded to eltrombopag and other TPO-RAs have demonstrated that thrombopoietic therapy can correct the monocyte activation defect in regulatory B cells (Bregs) that are functionally impaired in chronic ITP [22, 23]. Additionally, in ITP, antiplatelet antibodies produced by the immune system label platelets, promoting their recognition and phagocytosis by macrophages [5]. However, the precise mechanisms by which eltrombopag interacts with aging-related processes, such as immunosenescence and cell cycle regulation, remain poorly understood.

This study utilized transcriptomic and single-cell sequencing data to identify CDKN1A on macrophages as a potential therapeutic target of Eltrombopag in ITP patients. Furthermore, our findings suggest that activated macrophages may promote the progression of ITP through functional communication with transitional B cells. Interestingly, a subset of macrophages appears to exert their effector functions by differentiating into specialized subtypes that operate independently, without direct interaction with other immune cells. Overall, our results provide novel insights into potential diagnostic and therapeutic targets for ITP.

## Methods

### Data acquisition

The gene expression data for this study were obtained from the publicly available Gene Expression Omnibus (GEO) database under accession number GSE112278 and GSE202127. And single-cell RNA sequencing (scRNA-seq) data for this study were obtained from GEO database under accession number GSE196676.

## Differentially expressed genes (DEGs) analysis

DEGs analysis was performed using the DESeq2 package in R. The analysis was conducted at a significance level of *p* < 0.05, with False Discovery Rate (FDR) correction using the Benjamini-Hochberg method. A gene was considered significantly differentially expressed if the|log_2_ fold change (log_2_FC)| > 1 and adjusted p-value (FDR) < 0.05. Volcanos were used to visualize the results of the differential expression analysis.

## Functional enrichment analysis

Gene Ontology (GO) pathway enrichment analyses were performed using the clusterProfiler package in R. The significance threshold for GO enrichment was set to adjusted p-value < 0.05.

## Weighted gene co-expression network analysis (WGCNA)

WGCNA was performed using the GSE112278 dataset to identify gene modules associated with clinical traits. Genes with the top 50% variance were included for analysis. A soft-thresholding power (β) was determined to achieve a scale-free topology (R² ≥ 0.9), and an adjacency matrix was constructed and transformed into a topological overlap matrix (TOM). Gene modules were identified via hierarchical clustering and dynamic tree cutting, with a minimum module size of 50 genes. Modules with similar expression patterns were merged based on eigengene correlations ( ≥ = 0.8).

Module-trait relationships were assessed by correlating module eigengenes with clinical traits. Hub genes were identified based on module membership (MM ≥ 0.8) and gene significance (GS ≥ 0.2). Functional enrichment, including KEGG pathway analysis, was conducted using the “clusterProfiler” package. Protein-protein interaction (PPI) networks were visualized with the STRING database and Cytoscape.

## scRNA-seq analysis

The raw scRNA-seq data were processed and analyzed using the Seurat package (version 4.0) in the R statistical computing environment. Initially, the data underwent quality control (QC) steps to filter out low-quality cells. To ensure data quality, we filtered out low-quality cells based on stringent criteria: nFeature_RNA < 500 and percent_mito > 15%. Subsequently, the filtered data were imported into Seurat (version 5.2.0), where the SCTransform. method was applied for data normalization to correct for sequencing depth and cell cycle effects. Principal component analysis (PCA) was then conducted to reduce data dimensionality and extract the main sources of variation. Using Seurat’s *FindNeighbors()* function, a cell neighborhood graph was constructed based on the first 20 principal components to capture cell similarities. Cell clustering was performed with the *FindClusters()* function at a resolution of 0.4 to identify cell populations with similar transcriptomic profiles. For visualization of clustering results in a low-dimensional space, UMAP embeddings were computed using the first 30 PCA dimensions as input. To accurately annotate cell types for each cluster, we employed a combination of multiple classic cell type-specific marker genes. Additionally, to assess differentially expressed genes between cell populations, we utilized the *FindMarkers()* in Seurat, with the default Wilcoxon rank-sum test for statistical analysis.

### Pseudotime analysis

Pseudotime trajectories were calculated using CytoTRACE, which estimates the differentiation potential of cells. Higher CytoTRACE scores represent earlier stages of differentiation. Monocle2 was then used to visualize and order cells along a pseudotime axis based on their gene expression profiles, revealing differentiation paths within key immune cell populations. The expression of CDKN1A was analyzed along the pseudotime trajectory to assess its role in immune cell differentiation. The relationship between CDKN1A expression and differentiation status was examined for different immune cell types. Pseudotime trajectories were visualized using heatmaps and trajectory plots. Boxplots were used to compare CytoTRACE scores across cell types.

## Cell-cell communication analysis

To investigate cell-cell communication networks, we employed the CellChat package (version 1.1) to analyze potential ligand-receptor interactions between cell types. The CellChat algorithm uses curated ligand-receptor interaction databases to predict communication pathways between cell populations based on gene expression data. Significant cell-cell communication pathways were identified by analyzing the number and strength of ligand-receptor interactions within the dataset. We further visualized communication networks using graph-based methods to depict interactions between different cell populations.

## Molecular docking

To analyze the binding affinity between Eltrombopag and CDKN1A (p21), molecular docking simulations were performed using AutoDock. First, the 3D structure of ETP was retrieved from ChemSpider and optimized using Avogadro software by removing redundant hydrogen atoms and adjusting the 3D conformation. The 3D structure of CDKN1A was obtained from the Protein Data Bank (PDB ID: 7KYQ), and water molecules and co-crystallized ligands were removed. Hydrogen atoms were added, and partial charges were calculated using AutoDockTools. Molecular docking was conducted using AutoDock Vina. The docking results were analyzed using PyMOL and Discovery Studio, focusing on hydrogen bonds, hydrophobic interactions, and electrostatic interactions. The lowest binding energy pose was selected for further analysis.

### Macrophage phagocytosis assay

RAW264.7 macrophages were transfected with either a CDKN1A-overexpression plasmid or CDKN1A-targeting shRNA using Lipofectamine 3000 (Invitrogen), according to the manufacturer’s protocol. Corresponding negative controls were included, including empty vector for the overexpression group and non-targeting shRNA (shNC) for the knockdown group. For the phagocytosis assay, mouse-derived platelets were isolated from freshly collected mouse plasma and labeled with carboxyfluorescein diacetate (CFDA). Transfected macrophages were co-cultured with CFDA-labeled platelets at a ratio of 1:50 (macrophages: platelets) for 2 h at 37 °C in a humidified incubator. After incubation, cells were fixed with 4% paraformaldehyde, permeabilized, and stained with anti-F4/80 antibody (macrophage marker), followed by a fluorescent secondary antibody. Nuclei were counterstained with DAPI. Fluorescence images were captured using a confocal microscope (Leica, Germany).

### Statistical analysis

All statistical analyses were performed using R (version 4.3.1). To assess the normality of the data, the Shapiro-Wilk test was conducted. In instances where the data failed to meet the criteria for normal distribution, non-parametric tests were employed. For datasets that adhered to a normal distribution, the results were presented as the mean ± standard deviation (SD). Subsequent to this, an evaluation of the homogeneity of variances across all groups was carried out. Upon confirmation of homogeneity of variances, a one-way analysis of variance (ANOVA) was utilized. In the event of heteroscedasticity, Dunnett’s T3 test was applied. For data involving repeated measures, repeated measures ANOVA was implemented. A p-value threshold of less than 0.05 was established to determine statistical significance.

## Results

### Identify biomarkers that affect the response to Eltrombopag in ITP patients

**Fig. 1:**
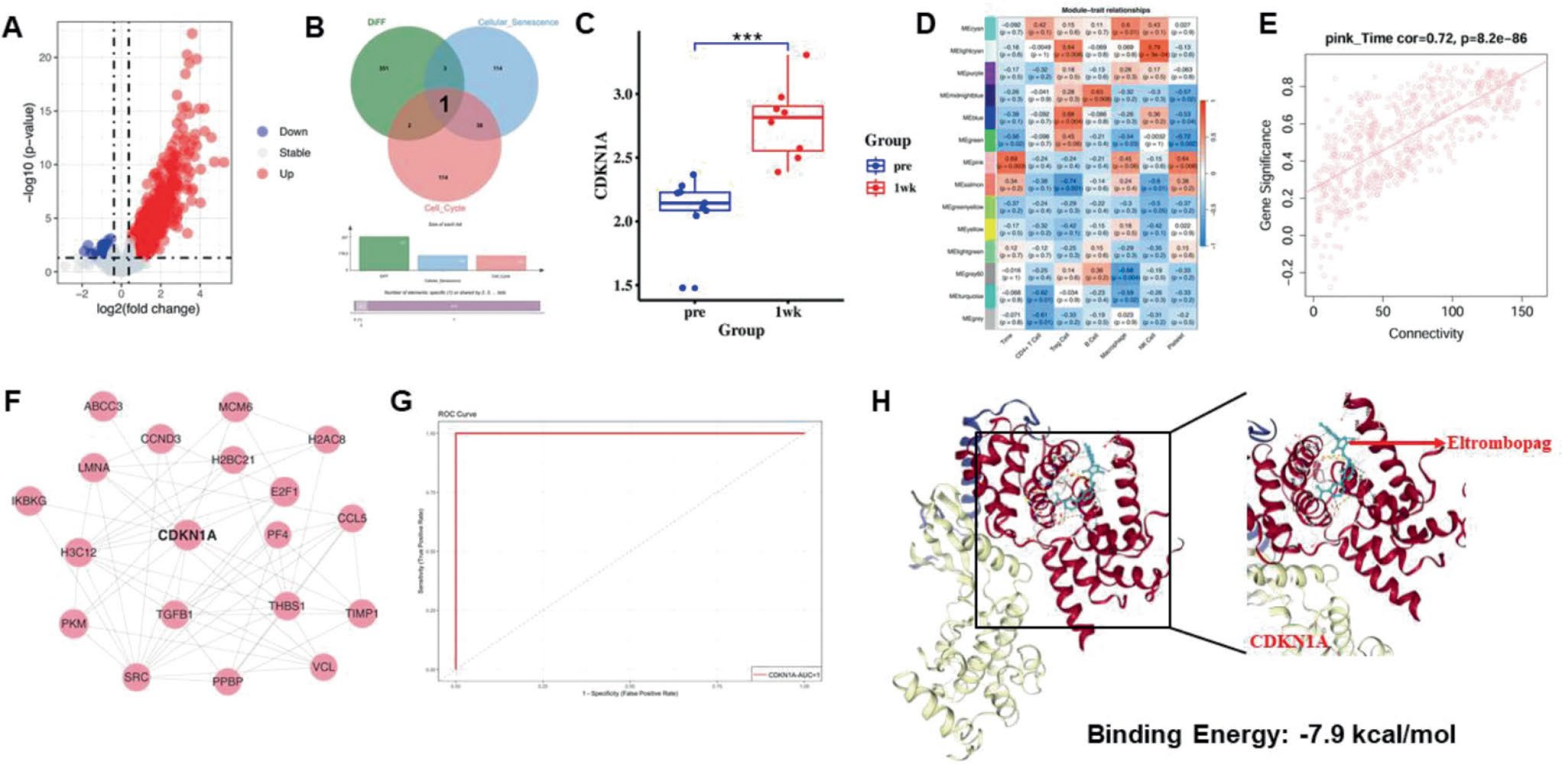
Identification of key genes influencing Eltrombopag treatment in ITP. **A** The volcano plot illustrates the results of differential gene analysis from the dataset (GSE112278) comparing ITP patients before and one week after Eltrombopag treatment **B** Venn diagram of DEGs intersecting with genes related to the cell cycle and cellular senescence **C** Expression of CDKN1A in ITP patients before and one week after Eltrombopag treatment **D** The module-feature relationships between module characteristic genes and clinical features in ITP patients. Red indicates positive correlations, while blue indicates negative correlations **E** Connectivity analysis between hub genes in the pink module and clinical features F. The PPI network of **G** ROC curve of CDKN1A for evaluating its diagnostic potential in ITP **H** Molecular docking results between Eltrombopag and CDKN1A, the binding energy between Eltrombopag and CDKN1A is -7.9 kcal/mol

We first conducted a DEGs in the ITP patient cohort within the GSE112278 dataset to identify key genes that may influence the therapeutic efficacy of Eltrombopag. CDKN1A was ultimately identified as a candidate gene through intersection analysis of the screened DEGs with gene sets related to cellular senescence and the cell cycle (Fig. 1A-B). Further analysis revealed that the expression level of CDKN1A in ITP patients was significantly upregulated following Eltrombopag treatment (Fig. 1C). Additionally, we employed weighted gene co-expression network analysis (WGCNA) to identify gene co-expression modules associated with clinical characteristics in the GSE112278 dataset (**Fig. S1A-C**). The results demonstrated that the pink module was significantly positively correlated with treatment duration (*p* = 0.003) and platelet count (*p* = 0.008), indicating its strong association with favorable prognosis (Fig. 1D). Connectivity analysis further revealed that the hub gene (mem-hub) within the pink module exhibited a strong correlation with clinical characteristics (Fig. 1E). KEGG enrichment analysis revealed that mem-hub genes were prominently involved in signaling pathways such as platelet activation, focal adhesion, and tight junctions (**Fig. S1D**), and a PPI network centered around CDKN1A was subsequently constructed (Fig. 1F).

To assess the potential diagnostic value of CDKN1A in ITP patients treated with Eltrombopag, we generated a receiver operating characteristic (ROC) curve using the GSE112278 dataset. The resulting area under the curve (AUC) value was 1.0, suggesting excellent classification performance between responders and non-responders (Fig. 1G). However, given the small sample size of this dataset, we sought to provide further supporting evidence. We next analyzed the GSE202127 dataset, which profiles mesenchymal stem cells (MSCs) treated with different concentrations of Eltrombopag. As shown in **Supplementary Figure S1E**, CDKN1A expression was significantly upregulated in cells treated with 15 µM Eltrombopag compared to controls (*P* = 0.047), and a further increasing trend was observed in the 50 µM group (*P* = 0.082). Although this model does not directly involve ITP samples, the dose-dependent regulation of CDKN1A expression implies its pharmacodynamic relevance to Eltrombopag exposure. In addition, molecular docking analysis revealed a favorable interaction between Eltrombopag and CDKN1A, with a calculated binding energy of − 7.9 kcal/mol (Fig. 1H). Therefore, our study demonstrated that CDKN1A may serve as an important biomarker for predicting ITP patients’ response to Eltrombopag treatment, further highlighting its potential and exceptional performance in the diagnosis of ITP.

### CDKN1A regulates ITP progression by affecting macrophage function

**Fig. 2:**
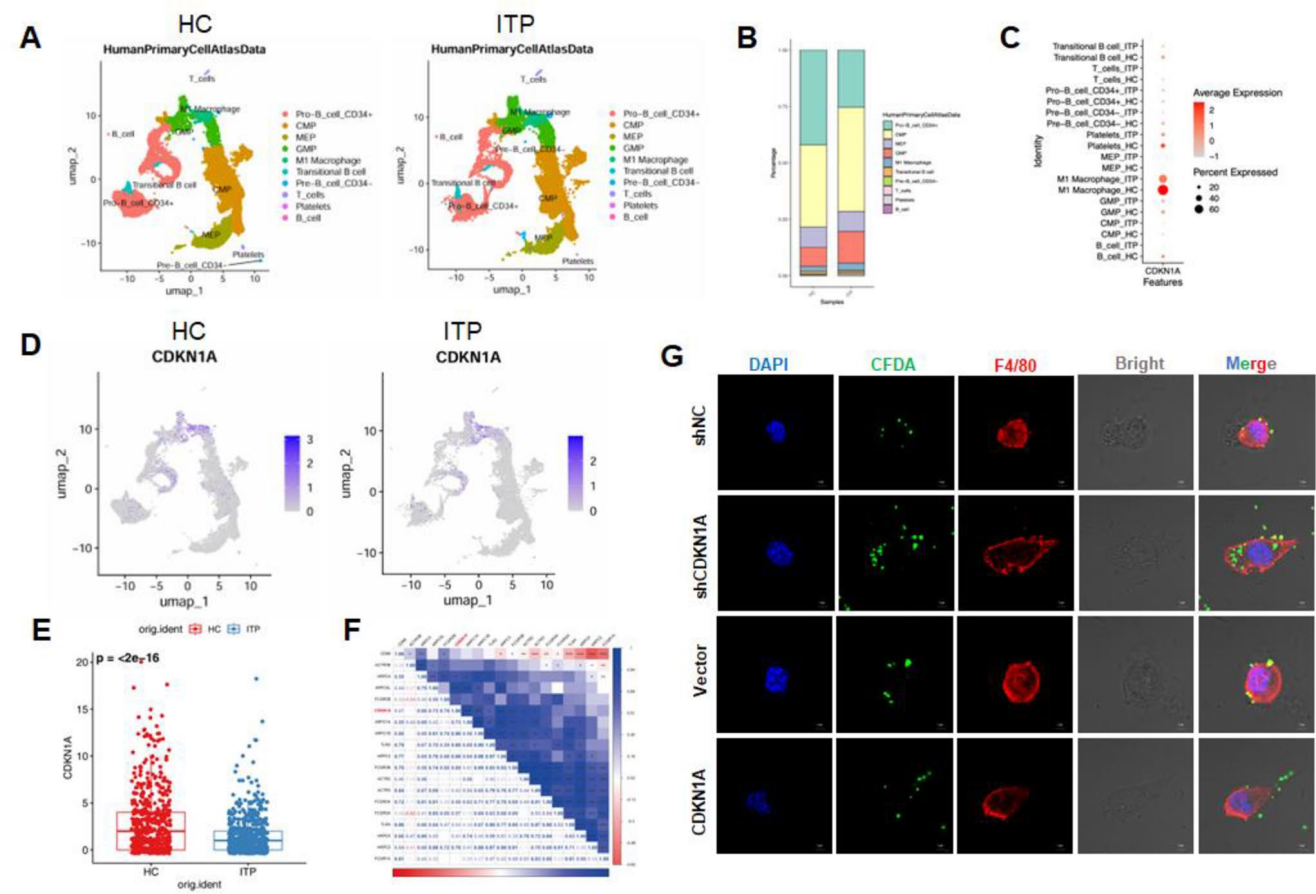
Downregulation of CDKN1A in macrophages of ITP patients **A** The t-SNE plot showing the cell annotation results based on the scRNA-seq dataset (GSE196676) of BM CD34^+^ HSPCs from four newly diagnosed treatment-naïve ITP patients and four healthy donors. MEP: Megakaryocyte-Erythroid Progenitor; GMP: Granulocyte-Macrophage Progenitor; CMP: Common Myeloid Progenitor **B** Proportions of different cell types in HC and ITP samples **C** Bubble plot showing CDKN1A expression in different cell types of HC and ITP **D** t-SNE plot depicting the distribution of CDKN1A across various cell types in HC and ITP **E** Expression of CDKN1A in macrophages from HC and ITP **F** Correlation analysis of CDKN1A with macrophage activation and phagocytosis-related proteins. Blue indicates positive correlations, while red indicates negative correlations **G** Immunofluorescence images showing phagocytosis of CFDA-labeled platelets (green) by RAW264.7 macrophages following CDKN1A knockdown or overexpression. Macrophages were stained with F4/80 (red) and nuclei with DAPI (blue). The shNC and vector groups served as respective controls for knockdown and overexpression. Scale bar = 10 μm

To elucidate the role of CDKN1A in ITP at the single-cell level, we further analyzed the single-cell dataset GSE196676. After QC, we annotated a total of 10 cell types, including Pro-B cells CD34^+^, CMP, MEP, GMP, M1 macrophages, transitional B cells, Pro-B cells CD34^−^, T cells, platelets, and B cells (Fig. 2A). Analysis of the changes in the proportions of each cell type in HC and ITP patients revealed that, compared to the HC group, the proportion of Pro-B cells CD34^+^ was significantly reduced in ITP patients, while the proportions of CMP, GMP, and M1 macrophages were significantly increased (Fig. 2B). Notably, CDKN1A was significantly enriched in M1 macrophages in both the HC and ITP groups, but its expression was significantly lower in ITP macrophages compared to those in the HC group (Fig. 2C-E). Additionally, correlation analysis revealed that CDKN1A was positively correlated with FcγR-mediated phagocytic function and macrophage phagocyte activation (Fig. 2F). To validate the regulatory role of CDKN1A in macrophage function, we constructed both overexpression and knockdown models in RAW264.7 cells. After confirming transfection efficiency, we assessed phagocytosis using fluorescently labeled platelets. As shown in Fig. 2G, silencing CDKN1A significantly enhanced macrophage phagocytic activity, while overexpression of CDKN1A suppressed platelet engulfment. Therefore, we hypothesize that the reduced expression of CDKN1A in ITP macrophages may enhance macrophage phagocytic function by modulating biological pathways such as the cell cycle, thereby promoting ITP progression.

### The differentiation and functional dynamics of M1 macrophages in ITP

**Fig. 3:**
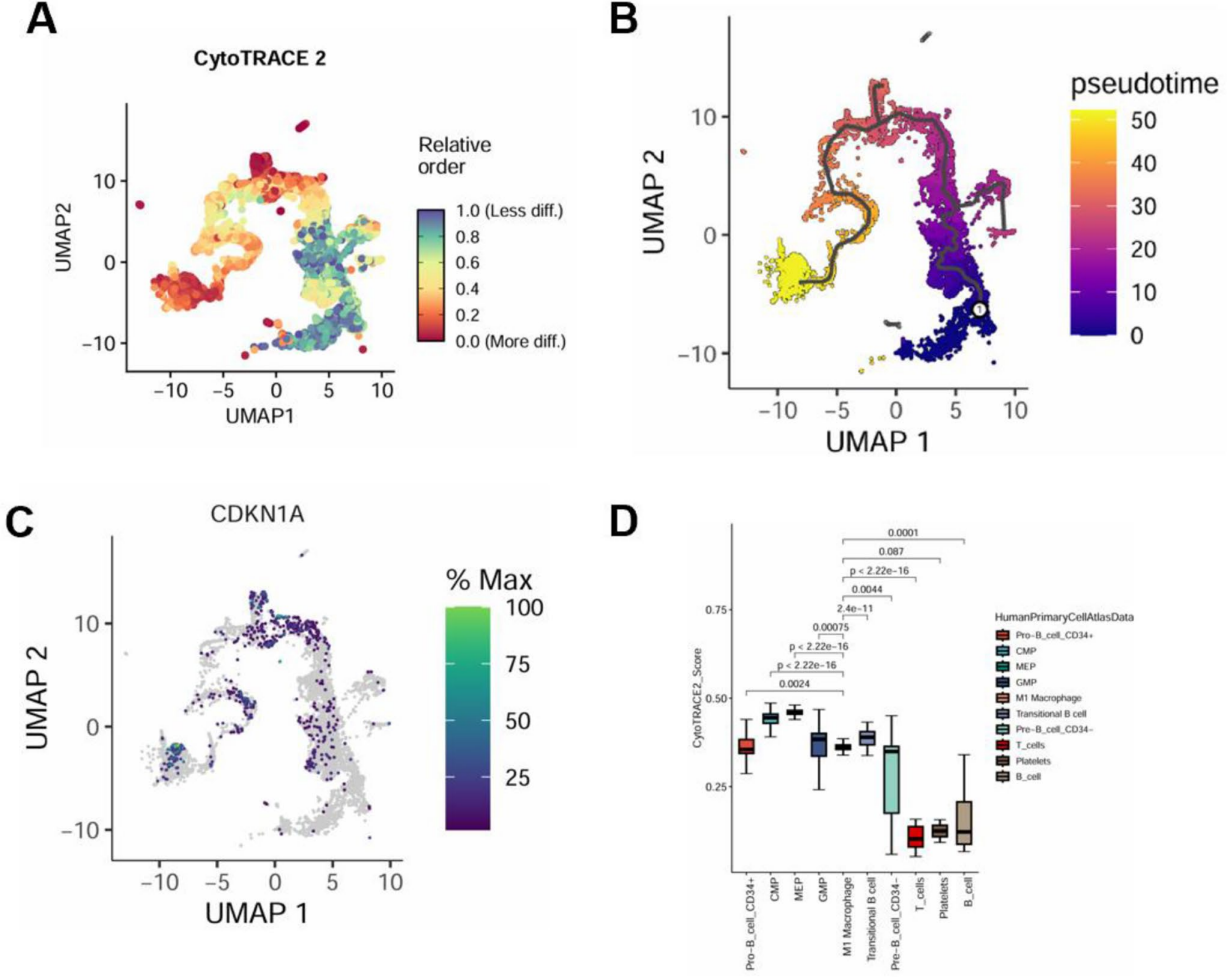
Pseudotime analysis of immune cell differentiation and functional states in ITP **A** CytoTRACE analysis of differentiation trajectories of various cells in ITP. Combined use of CytoTRACE and Monocle2 predicts the origin of M1 macrophages (left) **B** Pseudotime distribution plot illustrating the “temporal” sequence of differentiation of various cells in ITP **C** Expression distribution map of CDKN1A **D** Boxplot comparing CytoTRACE scores among different cell types

To trace the differentiation trajectory and dynamic alterations of macrophages in ITP, we conducted pseudo-time series analysis (Fig. 3A). The results revealed that CMP, GMP, and MEP, as bone marrow progenitor cells, exhibited high differentiation potential and were predominantly located at the initiation phase of differentiation. T cells, B cells, and platelets have differentiated into relatively mature cell types, nearing their final functional state, and are predominantly located at the final stages of differentiation (Fig. 3B). Given that CDKN1A may mediate ITP progression by regulating M1 macrophage function (Fig. 3C), we further compared the cytoTRACE scores between macrophages and other cell types. The results demonstrated that the difference in cytoTRACE scores between M1 macrophages and CMP, MEP, T cells, and transitional B cells was most significant (Fig. 3D). The higher cytoTRACE scores of MEP and CMP cells may reflect that they remain in an activated or proliferative state, attempting to compensate for immune microenvironment changes induced by thrombocytopenia by increasing proliferation. The lower cytoTRACE score of T cells suggests that they are more mature in ITP and may be in a more stable immune response state, though their role in the immune response warrants further investigation. The elevated cytoTRACE score of transitional B cells indicates that they may be in a more active or unstable immune state in ITP, potentially aggravating the pathological process by participating in the production of antiplatelet antibodies. These results demonstrate that significant differences exist in the differentiation and functional statuses of various cell types in ITP, particularly in cytoTRACE scores between M1 macrophages and CMPs, MEPs, T cells, and transitional B cells. The lower cytoTRACE score of M1 macrophages reflects their maturity and stability in immune responses, whereas the elevated cytoTRACE score of transitional B cells suggests that they may be in an unstable and active state in ITP, which may further exacerbate ITP progression through immune responses or by promoting the production of antiplatelet antibodies.

### CDKN1A mediates ITP progression through TGFβ signaling pathway

**Fig. 4:**
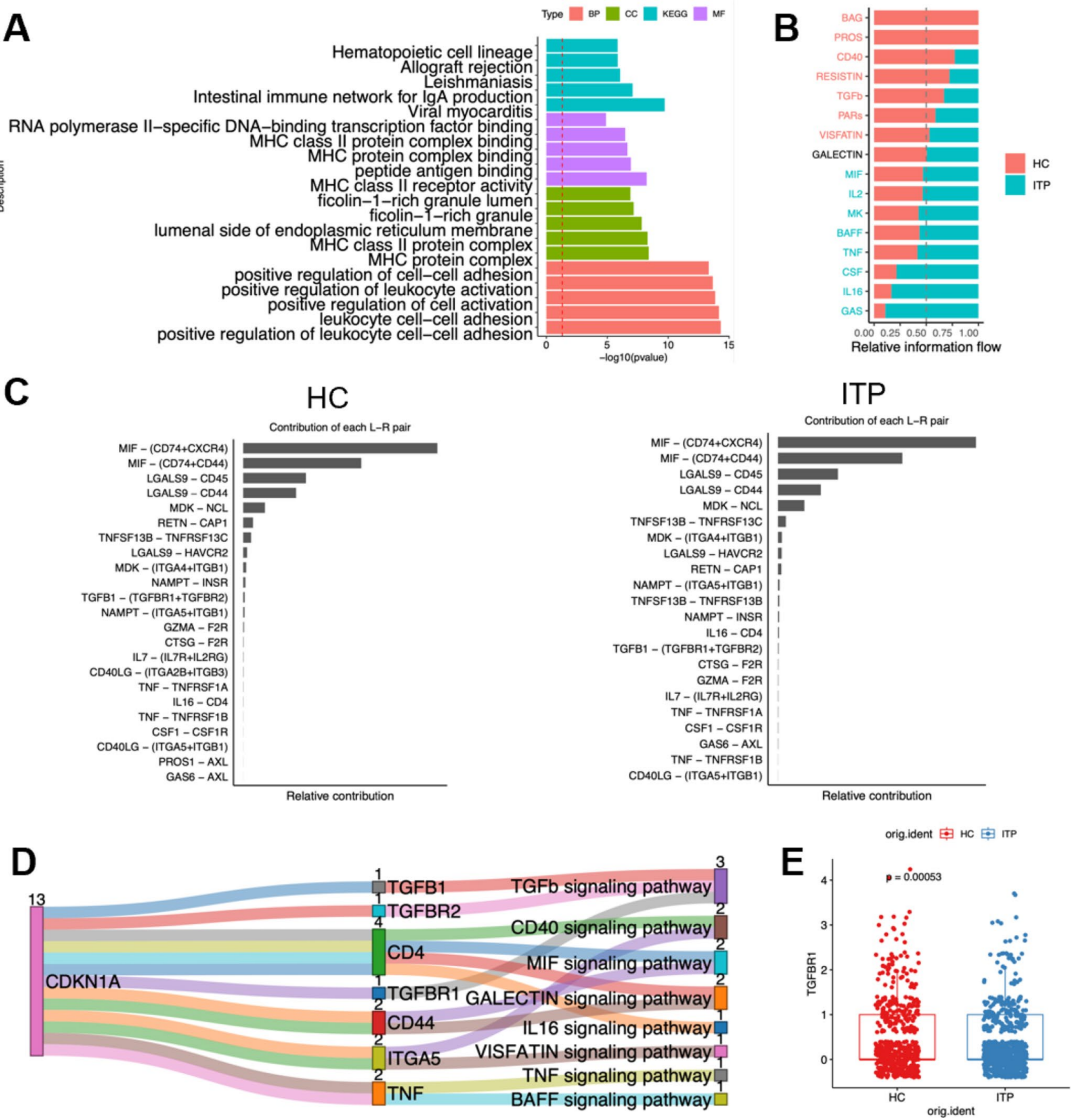
CDKN1A regulates macrophages via the TGFβ signaling pathway **A** GO analysis showing the enriched biological pathways of DEGs in macrophages **B** Differential signaling pathways in HC and ITP patients **C** List of receptor-ligand contributions in HC and ITP patients **D** Sankey diagram showing the interaction between CDKN1A and receptor-ligand pairs **E** Expression of TGFBR1 in macrophages from HC and ITP patients

Additionally, we conducted enrichment analysis on functions related to M1 macrophages. The results indicated that the differentially expressed transcription factors in M1 macrophages were closely associated with various immune-related biological processes, suggesting their critical roles in immune response, inflammatory response, antigen presentation, and leukocyte activation (Fig. 4A). We then analyzed the differentially enriched pathways in HC and ITP samples, investigating the contribution of ligand-receptor interactions in each pathway (Fig. 4B). Through screening the interacting proteins of CDKN1A via the PPI network, we identified a strong interaction between CDKN1A and proteins involved in the TGFβ signaling pathway (Fig. 4C-E). Notably, the expression of TGFβR1 in ITP was significantly lower than in the HC group, which was consistent with the expression changes of CDKN1A (Fig. 4F). Thus, our results suggest that CDKN1A may mediate ITP progression through the TGFβ signaling pathway in macrophages.

### Macrophages communicate with transitional B cells through TGFβ signaling

**Fig. 5:**
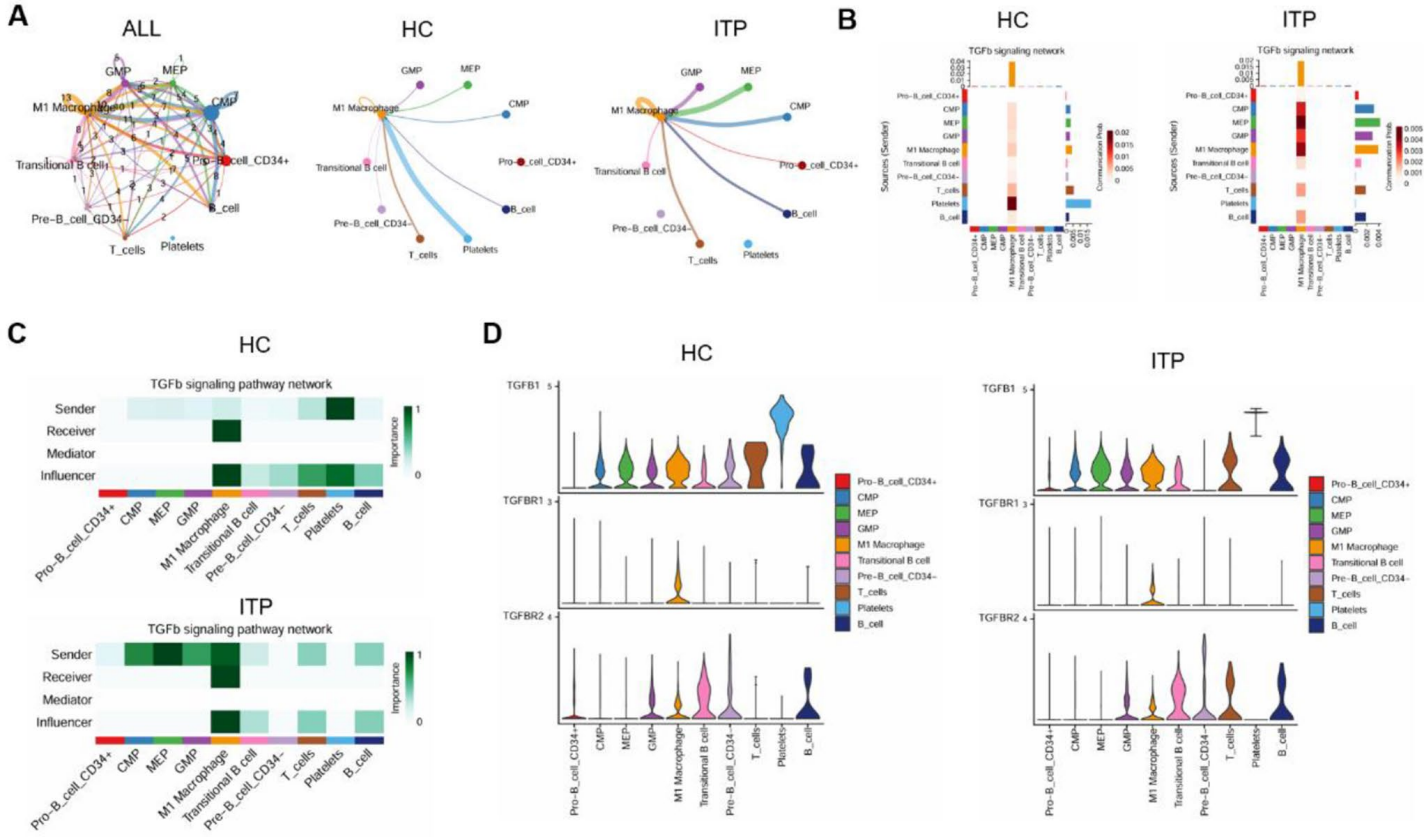
Macrophage communication with transitional B cells **A** Communication network between macrophages and other cell types **B** Heatmap of interaction strength between cell types in the TGFβ signaling pathway **C** Heatmap of roles of cell types in the TGFβ signaling pathway **D** Violin plot showing the expression of TGFB1, TGFBR1, and TGFBR2 in different cell types

To further investigate the interactions between macrophages and other cell types, we employed CellChat to analyze intercellular communication. The results revealed that the interactions between macrophages and GMP, MEP, CMP, transitional B cells, and B cells were significantly enhanced in ITP (Fig. 5A). Given that MEP, GMP, and CMP are early precursor cells in the hematopoietic stem cell lineage, they primarily contribute to blood cell production rather than the regulation of immune responses. Consequently, we focused on the connection between macrophages and transitional B cells. We then analyzed the role of transitional B cells and macrophages in TGFβ signaling. In contrast to HC samples, in ITP patients, apart from the connections between macrophage precursor cells (e.g., CMP, MEP, GMP) acting as “senders” and macrophages, B cells (including transitional B cells) also functioned as “senders,” while macrophages acted as “receivers” in the transmission of TGFβ signals (Fig. 5B-D). These results suggest that transitional B cells function as senders and macrophages as receivers, activating macrophage phagocytic function via TGFβ signaling, thereby promoting ITP progression.

### Functional changes of macrophages and transitional B cells in ITP patients

**Fig. 6:**
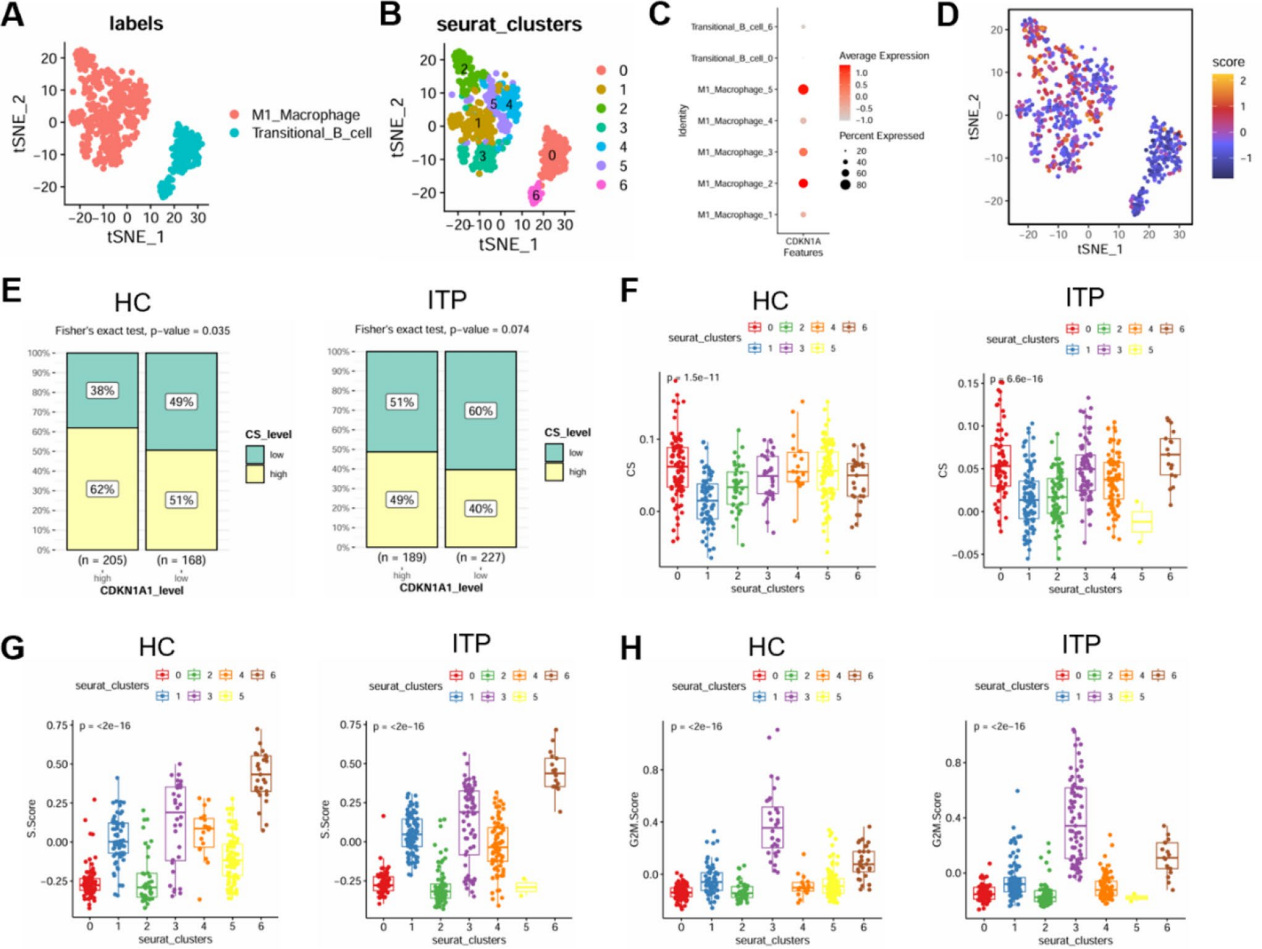
Functional characteristics of macrophages and transitional B cells **A**-**B** Reclustering analysis of M1 macrophages and transitional B cells, visualized using tSNE dimensionality reduction **C** Bubble chart showing the enrichment of CDKN1A across various cell types **D** tSNE plot illustrating the distribution of CDKN1A within different cell populations **E** Comparison of the correlation between CDKN1A and senescence in HC and ITP samples **F**-**H** Boxplots depicting the senescence and cell cycle scores for each subcluster in HC and ITP samples

To gain a deeper understanding of the functional characteristics of macrophages and transitional B cells in ITP progression, we conducted subcluster analysis on these cell types and identified two clusters of transitional B cells and five clusters of macrophages (Fig. 6A-B). Among the two transitional B cell subclusters (clusters 0 and 6), we observed distinct transcriptional profiles (**Fig. S2A**). Cluster 0 exhibited high expression of genes such as LINC01013, IGHD, ISG20, and MDM2, which are associated with B cell receptor activity and immune activation. In contrast, cluster 6 was enriched for GINS2, TYMS, MCM4, and GMNN, genes involved in DNA replication and cell proliferation. These findings suggest that the two transitional B cell subpopulations may represent different activation or differentiation states, potentially reflecting functional heterogeneity in the context of ITP.

Notably, CDKN1A expression was found to be significantly enriched in clusters 2 and 5 (Fig. 6C-D). As one of the markers of aging, higher aging levels in healthy samples are typically associated with increased CDKN1A expression. However, in ITP samples, we observed that high CDKN1A expression was associated with a slight reduction in aging levels, whereas low CDKN1A expression correlated with a more significant decrease in aging levels (Fig. 6E). This suggests that decreased CDKN1A expression in ITP patients may promote disease progression by maintaining cell viability. The relationship between each cell clusters, aging, and the cell cycle was further analyzed. Cluster 5, enriched in CDKN1A, has the lowest aging score (CS score) among all clusters, whereas the CS score of clusters 2 remained unchanged between healthy controls and ITP samples (Fig. 6F). By comparing the S-phase scores of each cell cluster in healthy samples and ITP patients, we found that the S-phase activity of cluster 5 was further reduced, suggesting that cluster 5 maintains low proliferation activity in healthy individuals, whereas in ITP patients, its proliferation activity is further inhibited due to the pathological state (Fig. 6G-H). This implies that macrophages in ITP may focus more on performing effector functions, such as platelet phagocytosis, rather than self-renewal.

**Fig. 7:**
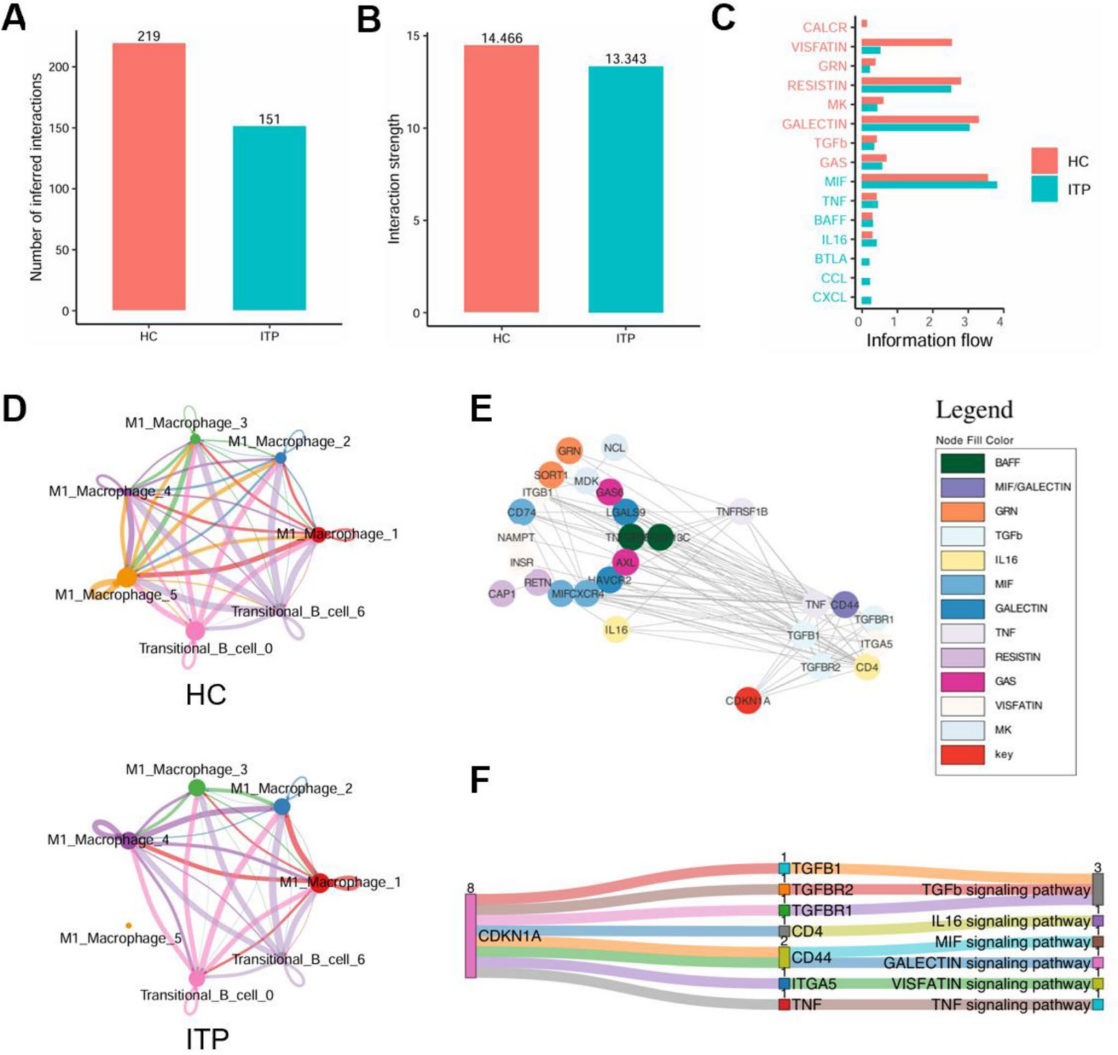
Macrophage subsets in ITP patients perform phagocytic functions independently of intercellular interactions. **A** Bar chart showing the number of pathways in HC and ITP **B** Bar chart displaying pathway weights in HC and ITP **C** Differential communication results between HC and ITP **D** Communication analysis between each subcluster in HC and ITP **E** PPI network illustrating the interactions between CDKN1A and ligands/receptors in signaling pathways **F** Sankey diagram showing the interactions between CDKN1A and ligands/receptors

Next, we analyzed the differentially expressed genes of each cell cluster and conducted KEGG pathway enrichment analysis to identify the molecular characteristics of each group of cells (**Fig. S2B-D**). Interestingly, the frequency and strength of cell communication across the seven clusters in ITP were lower than those in the healthy control group, indicating a communication defect between macrophages and transitional B cells in ITP (Fig. 7A-C). Specifically, the interaction between cluster 5 and other clusters in ITP was completely lost, resulting in the disruption of immune crosstalk (Fig. 7D). Through PPI network analysis, we further identified the interacting proteins of CDKN1A (Fig. 7E-F). Consistent with the previous results, CDKN1A exhibited a strong association with the TGFβ signaling pathway. Taken together, our results suggest that, during ITP progression, macrophages may differentiate into a specific subset capable of functioning independently of transitional B cell activation to promote platelet phagocytosis.

## Discussion

ITP is an acquired autoimmune hemorrhagic disorder in which the loss of immune tolerance plays a critical role in its pathogenesis [4]. Among these, megakaryocytes and macrophages are key immune cells involved in a variety of physiological and pathological processes [24, 25]. Eltrombopag, an oral thrombopoietin receptor agonist, increases platelet production by promoting the proliferation and differentiation of megakaryocytes [20]. It is used to treat chronic ITP patients who are refractory or intolerant to traditional therapies, particularly those unsuitable for splenectomy, and has demonstrated significant efficacy [21, 26, 27]. In our study, transcriptomic data screening revealed that CDKN1A expression was significantly downregulated in ITP macrophages. We further demonstrated that Eltrombopag may exert its therapeutic effect by targeting CDKN1A, underscoring its potential critical role in the treatment of ITP.

CDKN1A (also known as p21) is a critical regulator of the cell cycle [28]. The p21 protein encoded by CDKN1A inhibits Cyclin-CDK complex activity, preventing the transition from the G1 phase to the S phase [29]. It is regulated by p53 in the DNA damage response, playing a crucial role in cell cycle arrest and DNA repair [28, 30]. CDKN1A plays a significant role in tumorigenesis, aging, and metabolic diseases, and is thus considered a potential target for therapy and a prognostic marker [31–34]. Study has shown that CDDP/PEM-induced upregulation of CDKN1A can induce cell cycle arrest and ferroptosis, thereby promoting tumor cell death [35]. Additionally, Awatef Allouch et al. demonstrated that CDKN1A enhances the prognosis of T-ALL patients by increasing the phagocytic ability of tumor-associated macrophages through transcriptional inhibition of signal-regulatory protein α (SIRPα) [36]. Moreover, research has shown that inhibiting CDKN1A expression can prevent macrophage differentiation, thereby preserving their proliferative capacity [37].

In our study, CDKN1A was significantly upregulated following Eltrombopag treatment, and molecular docking analysis revealed a strong binding affinity between Eltrombopag and CDKN1A. Interestingly, single-cell RNA-seq analysis showed that CDKN1A expression was markedly reduced in macrophages from ITP patients, and its expression positively correlated with macrophage activation and FcγR-mediated phagocytosis pathways. To experimentally validate its functional role, we modulated CDKN1A expression in RAW264.7 macrophages and observed that CDKN1A knockdown significantly promoted platelet phagocytosis, while its overexpression suppressed phagocytic activity. These results provide direct evidence that CDKN1A negatively regulates macrophage phagocytosis, possibly by modulating their activation state or cell cycle status. Taken together, our findings suggest that Eltrombopag may restore platelet counts in ITP patients in part by modulating CDKN1A expression in macrophages, thereby suppressing their hyperactive phagocytic behavior. This study uncovers a novel immunomodulatory mechanism of Eltrombopag and positions CDKN1A as a potential therapeutic target in ITP.

Studies have shown that the pathogenesis of ITP is multifactorial [4]; however, immune dysregulation—particularly macrophage-mediated platelet clearance and the imbalance between effector and regulatory immune responses—remains central to disease progression [4, 38]. As critical components of the immune system, macrophages play a crucial role in maintaining platelet homeostasis [39, 40]. Under normal conditions, macrophages maintain immune tolerance and regulate the balance between immune activation and suppression [41]. In the context of ITP, however, macrophage-mediated clearance of antibody-coated platelets results in a decrease in platelet count, triggering the clinical manifestations of the disease [42, 43]. Furthermore, macrophages not only play a direct role in platelet clearance but also participate in the regulation of immune responses through the production of proinflammatory cytokines and the modulation of B cell activity [44]. Dysfunctional macrophages may exacerbate platelet destruction and contribute to the progression of chronic ITP. Therefore, targeting macrophage-related pathways to reduce platelet clearance has emerged as a major focus of research in the field of ITP. Recent studies suggest that quercetin may serve as a potential therapeutic agent for ITP by inhibiting platelet phagocytosis in M1 macrophages via its anti-inflammatory effects [45]. However, the mechanisms underlying the targeting of macrophage M1 polarization and the inhibition of its phagocytic function require further investigation.

In this study, single-cell analysis revealed a significant increase in the proportion of M1 macrophages in ITP patients compared to healthy controls. Notably, the expression of TGFBR1 in macrophages exhibited a downregulation trend similar to that of CDKN1A, suggesting that macrophage activation in ITP may be closely linked to dysregulation of the TGFβ signaling pathway. The TGFβ pathway is known to interact with multiple cellular processes, including ferroptosis, senescence, and cell cycle regulation. Previous studies have shown that TGFβ signaling can suppress cell cycle progression by upregulating CDK inhibitors, leading to cell cycle arrest and enhanced expression of aging-associated genes [46]. In addition, TGFβ signaling has been implicated in the regulation of ferroptosis through modulation of intracellular iron homeostasis and oxidative stress responses [47]. The interplay between TGFβ signaling, ferroptosis, and senescence may collectively influence macrophage proliferation, survival, and function in the ITP microenvironment [48]. Therefore, targeting TGFβ signaling in macrophages may offer a promising therapeutic approach to modulate immune activation and disease progression in ITP. However, although we identified TGFBR1 downregulation in ITP macrophages, the upstream regulatory mechanisms remain unclear. Further studies involving chromatin accessibility profiling (e.g., ATAC-seq) and transcription factor occupancy analysis will be necessary to delineate the epigenetic and transcriptional control of TGFBR1 expression under inflammatory conditions.

Further cell communication analysis revealed that the interaction between macrophages and transitional B cells was amplified in ITP. Transitional B cells represent a crucial stage in B cell development and play a pivotal role in immune tolerance and immune responses, particularly in the elimination of self-reactive B cells to prevent autoimmune diseases. Dysfunction of transitional B cells is strongly associated with several autoimmune diseases (e.g., systemic lupus erythematosus, rheumatoid arthritis) [49, 50]. Studies have demonstrated that the increased number of CD19^+^CD24^high^CD38^high^ B cells (transitional B cells) and pre-germinal center B cells reflects an activated autoimmune response and may predict the future treatment response in untreated ITP patients [51]. However, current research on the role of transitional B cells in ITP remains limited. Our results indicate that within the TGFβ signaling pathway, transitional B cells function as signal senders, activating the phagocytic capacity of macrophages as receivers, resulting in the loss of a substantial number of platelets and promoting disease progression. By re-clustering macrophages and transitional B cells to reveal their functional characteristics, we discovered that in ITP, macrophage subtype 5, which is significantly enriched for CDKN1A, does not interact with other cells and is closely associated with senescence and the cell cycle. This suggests that during the progression of ITP, macrophages may differentiate into independent subpopulations with specialized functions, becoming more inclined to perform effector functions, such as platelet phagocytosis, rather than participating in the coordination of immune cells. This feature may reflect the disruption of the immune microenvironment in ITP patients and suggests that CDKN1A plays a crucial role in regulating the cell state of this subpopulation, such as by inhibiting excessive proliferation and focusing on specific functions. In general, our study offers new insights into the interaction between macrophages and transitional B cells in ITP, addressing an important gap in this field.

## Conclusion

In summary, our study successfully identified CDKN1A, a key protein associated with ferroptosis, senescence, and the cell cycle, and highlighted its potential as a therapeutic target for Eltrombopag in the treatment of ITP. We observed a reduction in CDKN1A expression in macrophages from ITP patients, where it interacts with transitional B cells via the TGFβ signaling pathway, ultimately contributing to immune imbalance. These findings offer novel insights into the pathogenic mechanisms and potential diagnostic markers of ITP, while also suggesting new therapeutic avenues, thereby advancing our understanding of the disease.

## Electronic supplementary material

Below is the link to the electronic supplementary material.

Supplementary Material 1

## Data Availability

No datasets were generated or analysed during the current study.
