# Supplementary material for: CDKN1A as a potential target for Eltrombopag treatment in ITP and its regulation of the communication between macrophages and transitional B cells in ITP
Source: Ann Hematol. 2025 Jun 14;104(6):3183–97. doi: 10.1007/s00277-025-06436-5 (PMC12283840; doi:10.1007/s00277-025-06436-5)
Supplement: Supplementary file 1 — Supplementary Material 1 [file 277_2025_6436_MOESM1_ESM.docx]

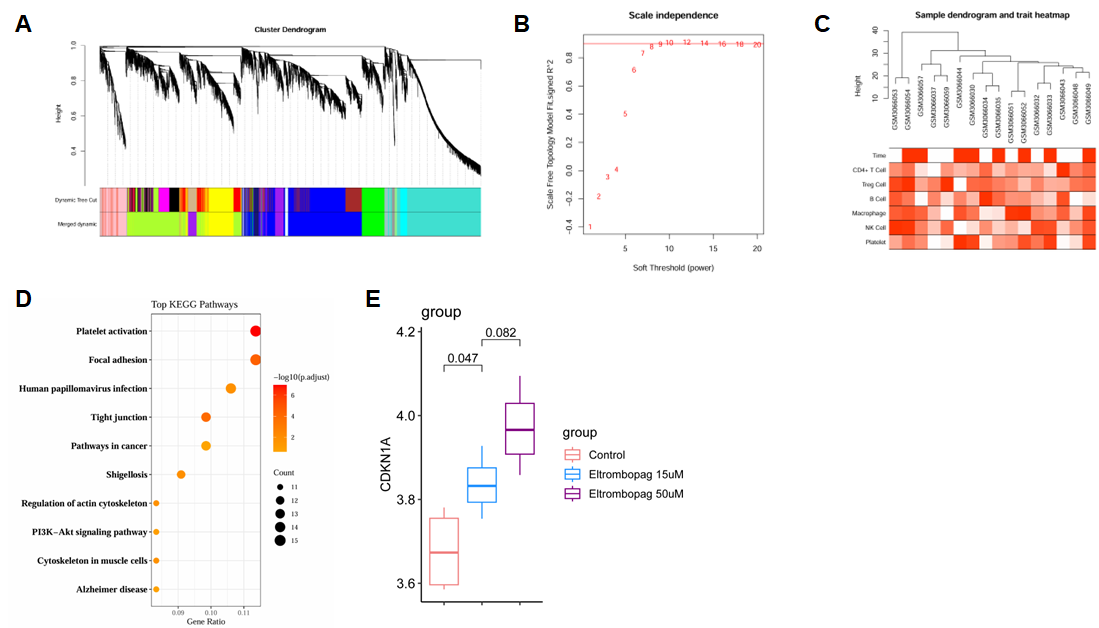


**Figure S1: Weighted gene co-expression network construction and identification of key modules**

A. Dendrogram showing hierarchical clustering results of ITP dataset (GSE112278). Each branch represents a cluster, with branch lengths reflecting the similarity or distance between clusters.

B. Soft-threshold power values based on scale independence and mean connectivity.

C. Cluster dendrogram of samples in the ITP dataset (GSE112278) based on expression patterns. Clinical information is indicated in red.

D. KEGG pathway enrichment bubble chart for hub genes in the pink module.

E. Box plot showing the expression levels of CDKN1A in mesenchymal stem cells treated with different concentrations of Eltrombopag (GSE202127).

**
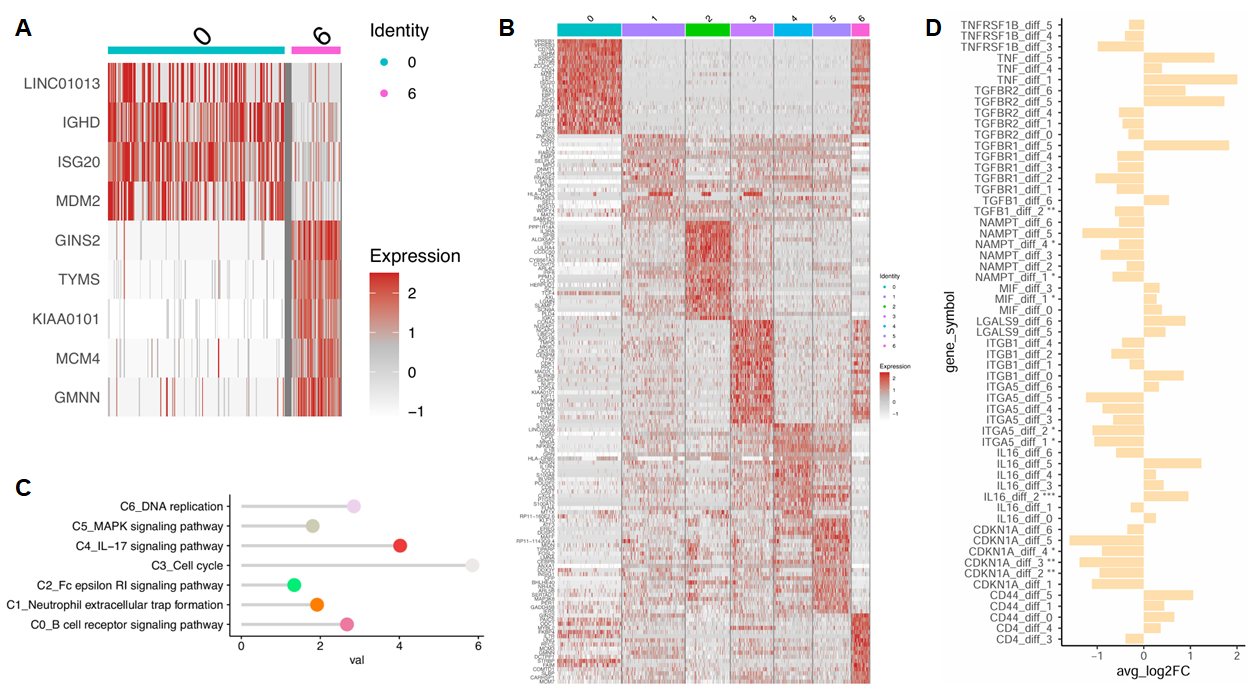
Figure S2: Subcluster differential analysis**
A. Heatmap showing representative differentially expressed genes between transitional B cell subclusters cluster 0 and cluster 6.

B. Heatmap of differential gene expression analysis across subclusters.

C. KEGG pathway results of the top 15 genes for each group.

D. Differences in receptor/ligand gene interactions between HC and ITP groups.
